# Efficacy of Oral Steroids for Acute Acoustic Trauma

**DOI:** 10.1159/000522051

**Published:** 2022-03-01

**Authors:** Elchanan Zloczower, Nir Tsur, Shir Hershkovich, Nir Fink, Tal Marom

**Affiliations:** ^a^Medical Corps, Israel Defense Forces, Tel Hashomer, Israel; ^b^Department of Otolaryngology-Head and Neck Surgery, Kaplan Medical Center, Rehovot, Hebrew University of Jerusalem, Jerusalem, Israel; ^c^Department of Otolaryngology-Head and Neck Surgery, Rabin Medical Center, Tel Aviv University, Petach Tiqva, Israel; ^d^Department of Communication Disorders, School of Health Sciences, Ariel University, Ariel, Israel; ^e^Department of Otolaryngology-Head and Neck Surgery, Samson Assuta Ashdod University Hospital, Faculty of Health Sciences, Ben Gurion University, Ashdod, Israel

**Keywords:** Sensorineural hearing loss, Tinnitus, Steroids, Acute acoustic trauma

## Abstract

**Objective:**

This study aimed to study the effect of steroid treatment on new-onset sensorineural hearing loss (SNHL) in subjects presenting shortly after an audiometry-confirmed acute acoustic trauma (AAT) injury.

**Study Design:**

This is a case-control study.

**Methods:**

We identified healthy military personnel who presented with AAT injury to the Israeli Defense Forces Medical Corps Otolaryngology/Audiology Services during 2016–2020. Patients were nonrandomly allocated to a treatment arm, where they received steroids (prednisone, 1 mg/kg, 60 mg maximal daily dose), administered for either ≥7 days or <7 days, or to a control arm, in which no treatment was offered besides loud noise avoidance. Audiometries were conducted within 7 days following the AAT and within 1 month later. We compared changes in bone conduction (BC) and air conduction (AC) thresholds at 2–8 kHz.

**Results:**

Of the 263 enrolled subjects, 137 (52%) received steroids and 126 (48%) received no treatment. Subjects who were treated early (<24 h) with high-dose steroids and for ≥7 days demonstrated significantly better hearing outcomes, compared with the nontreatment group. Subjects in the steroids group demonstrated 13–14 dB average improvement in BC thresholds at 3 and 4 kHz (*p* = 0.001) and additional 7–8 dB average improvement in AC thresholds at 6 and 8 kHz, compared with the nontreatment group (*p* < 0.0001). These observations were more compelling in patients who initially presented with worse hearing losses (>35 dB). No statistically significant differences were observed in AC/BC pure tone average between the two groups.

**Conclusions:**

Early oral steroids are recommended in AAT injuries and were shown to improve hearing outcomes within 1 month.

## Introduction

Acute acoustic trauma (AAT) injuries are common worldwide, especially in military settings, and can lead to a significant morbidity and impairment in quality of life [Hecht et al., 2019]. Noise-induced damage to the middle and inner ear includes tympanic membrane perforation, ossicular chain disruption, and injuries to the cochlea and the auditory/vestibular systems [Clark and Bohne, 1999]. As a result, hearing loss (usually sensorineural, SNHL), vertigo, and/or tinnitus may occur. While tympanic membrane perforations and middle ear injuries may occasionally occur, the classic presentation of acoustic trauma is SNHL in the presence of an intact tympanic membrane, with a typical decrease in hearing thresholds at 3-, 4-, 6-, and 8-kHz frequencies [Ryan et al., 2016]. The severity of an AAT injury is proportional to the noise intensity, distance from the noise source, use of hearing protective devices (HPDs), and the personal sensitivity, which is affected by the individual's genetic susceptibility [Konings et al., 2009]. Despite the high prevalence of AAT and the associated medical costs and economic burden [Alamgir et al., 2016; Garcia et al., 2018], currently there is no ideal agreed treatment protocol for AAT injuries in neither civilian nor military settings, in the absence of evidence-based knowledge.

The characteristics of noise emitted from weapons vary in respect to the caliber and ammunition type in the military setting. A 5.56-mm assault rifle and a 7.62-mm machine gun display a peak sound pressure level (SPL) of 152–154 dB, measured by using blast probe microphones at a distance of 0.5 m from the shooter [Nakashima et al., 2017], whereas a peak of ∼180 dB may be measured at the nearby vicinity of sniper rifles. Mortar and Grenade launcher display peak dozen pressures of 173-dB SPL and 175-dB SPL, respectively, measured in-the-ear. Free-field levels can be estimated as 8–12 dB lower than in-the-ear levels [Murphy et al., 2015].

Over the recent decades, various pharmacological treatments for AAT injury have been proposed and tried: steroids, vasodilators, antioxidants, and vitamins. Among them, oral steroids, with or without hyperbaric oxygen therapy (HBOT), were the focus of many studies, as they were shown to be the most effective treatment for AAT injuries [Choi et al., 2019; Koochakzadeh et al., 2020; Ahmed et al., 2021]. The rationale for HBOT in AAT injuries is based on experimental studies, showing that noise exposure results in cochlear hypoxia [Lamm and Arnold, 1996]. A few studies investigated the efficacy of HBOT in AAT injuries: Ylikoski et al. demonstrated significantly better audiometric results both in low and high frequencies in patients with AAT injuries who were treated with early-onset HBOT (within 3–4 days after injury), compared with those who did not receive HBOT at all [Ylikoski et al., 2008]. Furthermore, Oya et al. [2019] showed that a combination of oral steroids and HBOT significantly improved audiometric results together with alleviation of subjective symptoms (i.e., aural fullness and tinnitus), even when treatment was given 3–4 weeks after initial injury. However, the latter study did not compare between patients receiving only steroid treatment and patients treated with steroids and HBOT, and therefore the exact contribution of HBOT in AAT injury is not clear. A recent study by Holy et al. [2021] showed that a combination of oral steroid therapy and HBOT given within 7 days after AAT injury is superior to oral steroid treatment alone, with significant better audiometric results in low frequencies (500 Hz and 2,000 Hz). Yet, most studies and meta-analyses included only relatively small cohorts of subjects (sample size ranges from 37 to 108 subjects), with a high degree of divergence in respect to the mechanism of injury (i.e., different kinds of weapons or other explosives), and lacked a uniform treatment protocol and sufficient long-term follow-up period.

To date, there is no uniform treatment protocol for AAT injuries also in the Israel Defense Forces (IDF) Medical Corps. We previously described our experience in AAT injuries during operation “Operation Protective Edge” (Gaza war of 2014) and showed that oral steroids greatly improved hearing thresholds in subjects presenting with moderate and severe SNHL secondary to AAT [Yehudai et al., 2017]. Following our initial findings, we attempted to seek an ideal steroid treatment protocol, in respect to the greatest improvement of audiometric outcomes performed a few weeks after treatment. In this study, we sought to characterize the response to oral steroids given at different durations in a large cohort of subjects who presented shortly following an AAT injury in the IDF, but not at a wartime scenario.

## Subjects and Methods

### Setting

Subjects with suspected AAT injuries were referred as early as possible to the IDF ambulatory Audiology outpatient clinics within 1 week after the exposure to the loud acoustic stimulus, and in some cases, telemedicine consultations were given. Of note, some subjects were referred to civilian hospital clinics/emergency rooms, if the military facilities were unavailable. A formal otomicroscopy was performed and was supplemented with tuning forks examination (512/1,024 Hz). Then, subjects were sent to a comprehensive audiometry to ascertain audiometric proof of AAT injury or defer it and then were evaluated by an otolaryngologist for treatment decision purposes.

### Subjects

We identified military personnel who presented to our clinics between January 2016 and January 2020, with a history and physical examination that were consistent with an AAT injury (presentation after noise exposure <3 weeks) and had at least 2 audiometries within 3-month interval between them (mean: 19.95 ± 15.75 days after injury, range: 14–119 days, of which 93.5% were less than 30 days). We excluded (1) patients who had tympanic membrane perforations or other middle ear injuries, (2) referrals not related to noise exposure or other conditions associated with an abrupt hearing impairment irrespective to a recent noise exposure (e.g., sudden SNHL), (3) subjects with previous chronic ear disease or ear surgery, (4) subjects with a previously known hearing loss, (5) subjects with AAT injuries whose files were incomplete because only IDF clinic records were available for analysis, and (6) subjects with normal audiometry charts, despite an allegedly AAT injury. Overall, 263 subjects met these inclusion and exclusion criteria. Pre-AAT injury hearing thresholds of enrolled subjects were assumed to be normal, due to the lack of any documented previous hearing complaints, records, or tests before their draft to the IDF.

### Audiometry

Pure tone audiometry (PTA) was performed using the same Amplaid audiometer (model-AC40 two-channel audiometer). Unmasked and masked pure tone air conduction (AC) and bone conduction (BC) thresholds were recorded for each ear separately at 500 Hz, 1 kHz, 2 kHz, 3 kHz, and 4 kHz and for AC also at 6 kHz and 8 kHz.

Interpretation of audiometry was performed as follows:

1. Normal hearing: when AC and BC thresholds were ≤25 dB at all frequencies.
2. Conductive hearing loss: when ≥2 frequencies demonstrating an air-bone gap (ABG) >10 dB and no BC threshold >25 dB.
3. Sensorineural hearing loss (SNHL): presence of any threshold of ≥30 dB at any frequency and no more than one frequency with an ABG of ≥15 dB.
4. Mixed hearing loss (MHL): ≥2 frequencies demonstrating an ABG of ≥15 dB and ≥1 BC thresholds of ≥30 dB.

Mild, moderate, severe, and profound hearing losses were defined as hearing level thresholds between 25–39, 40–69, 70–90, and >90 dB in PTA, respectively [Clark, 1981]. We defined AAT injury if audiometry confirmed SNHL or MHL with an SNHL component of >30 dB in at least 3 consecutive frequencies.

PTA was calculated by averaging thresholds at 500 Hz, 1 kHz, 2 kHz, and 4 kHz. Thresholds for 500-Hz, 1-kHz, and 2-kHz frequencies were termed “low-frequency thresholds,” and 3-kHz, 4-kHz, 6-kHz, and 8-kHz thresholds were termed “high-frequency” accordingly. This differentiation was used for determining the predominant hearing loss type and for follow-up purposes and assessment of any improvement. We categorically subdivided subjects according to their hearing loss severity as measured by average of air conduction thresholds between 500 Hz and 2 KHz at first audiometry: ≤35 dB and >35 dB.

There was a great variability in the presentation of our study patients who came from remote field units. When the first audiometry did not show signs of an AAT injury, as defined above, the audiologist concluded that the patient had normal hearing or transient threshold shift, and thus did not refer back the patient urgently to the otolaryngologist. However, at times when the audiometry was consistent with AAT injury, the patient was referred at the earliest convenience to be seen by an otolaryngologist, and sometimes a telephonic consultation was performed with him for treatment decision purposes.

### Treatment Groups

Subjects were retrospectively allocated to one of the 2 treatment groups, according to the otolaryngologist's discretion and experience:

1. No medical treatment, but with recommendations for abstaining from loud noise exposure over the next few weeks, including participation in shooting activities.
2. Oral steroid treatment, together with proton pump inhibitors, with recommendations for abstaining from loud noise exposure over the next few weeks, including participation in shooting activities.

We observed 2 treatment patterns:

i. High-dose prednisone (1 mg/kg, 60 mg maximal daily dose) for ≥7 days and then tapering off over ≥7 days.
ii. High-dose prednisone (1 mg/kg, 60 mg maximal daily dose) for <7 days and tapering off over <7 days.

For analysis purposes, we combined those 2 groups of subjects who had received steroid treatment. Other treatments, such as intra-tympanic steroid injections, HBOT, or other combinations, were not considered as first-line or standard treatments for AAT injury in our practice during the study years.

### Statistical Plan

Data were recorded using Microsoft Excel software. Statistical analyses were performed using IBM© SPSS© Statistics for Windows, Version 25. Contingency table analysis for comparing rates between unmatched samples was performed using the χ^2^ test or Fisher's exact test, as appropriate. The *t* test was used to compare scale variables, specific threshold, PTA, and differences in hearing thresholds for correlation of initial hearing loss with degree of recovery of hearing. The *t* test was also used to compare means of independent samples. All tests were considered significant if *p* values were ≤0.05. Analysis was performed according to the ear and patients. In patients presenting with bilateral AAT injuries, hearing thresholds of the worse ear were analyzed.

## Results

### Demographics

Of the 263 subjects presenting with ATT injury, 232 (88%) were males with a mean age of 20.15 ± 2.38 years (range 18.2–39.9 years). Of the total cohort, 126 (48%) subjects did not receive any steroid therapy, while 137 (52%) subjects received any of the 2 steroid therapy regimens. Overall, we did not notice any specific trends in the treatment protocol during the study years. Table 1 shows the clinical and demographic data of our study population. The most common symptom in the nonsteroid group was a new-onset tinnitus (76.2%), while the most common symptom in the steroid group was a new-onset hearing loss (72.3%), *p* = 0.037. For both groups, exposure to noise in an open setting (a shooting gallery without a rooftop) was similar.

### Improvement in Parameters

We overall compared hearing levels in 252 ears of patients who did not receive steroid treatment with 274 ears of patients who were treated with steroids. When evaluating improvement proportions, Table 2 shows that there was an overall hearing improvement of 30.3% in the treatment arm versus 21.4% in the nontreatment arm (*p* = 0.02). However, no statistically significant differences were observed regarding other improvement parameters.

### First Audiometry Variables

The mean BC PTA average was statistically higher among the steroid group, when compared with the nonsteroid group (16.7 vs. 12.98 dB, *p* < 0.0001). When we evaluated high-frequency PTA averages, these were statistically significantly lower among the nonsteroid group, when compared to the steroid group (25.41 vs. 33.91 and 24.9 vs. 31.37 dB, at 6 kHz and 8 kHZ, respectively, *p* < 0.0001).

### Second Audiometry Variables

The mean BC PTA average showed no statistically significant difference between the nonsteroid and the steroid group (10.98 vs. 12.5 dB, *p* = 0.102). However, there was a statistically significant difference in the high-frequency PTA average, with higher levels for the steroid group (for BC, at 3 kHz and 4 kHz, and for AC, at 6 kHz). Also, the differences between the steroid group and the nonsteroid group were significantly higher among the steroid group, measuring some 7–8 dB on average improvement after treatment (Table 3).

### Onset of Treatment

The mean time from AAT injury to the first audiometry was 5.4 ± 5.25 days for the nonsteroid group and 4.79 ± 7.86 days for the steroid groups (*p* = 0.467). The mean time from AAT injury to the first otolaryngologist examination was significantly longer among the nonsteroid group, compared with the steroid group (7.75 vs. 3.86 days, *p* < 0.0001). Initiating steroid treatment within 24 h after the AAT injury was associated with a better outcome as measured by improvement in 4-kHz BC thresholds and 6-kHz AC thresholds in the second audiometry when compared with initiating steroid treatment later than 24 h after the AAT injury (Table 4).

### Different Steroid Protocols

We compared different protocols of steroid treatment with variable durations of treatment. Longer duration of treatment (≥7 days) was associated with a better outcome than shorter duration protocols (<7 days) (Table 5).

## Discussion

The main goal of this study was to evaluate the effect of oral steroids in AAT injuries, since there is conflicting evidence in the literature and a lack of clear treatment protocol. Our results show that oral steroid treatment is correlated with better audiometric outcomes in subjects with AAT injuries. We found that early treatment (<24 h after injury) and long-term high-dose steroid treatment (prednisone 60 mg daily ≥7 days) was superior to late (>24 h after injury) and shorter duration treatment, as assessed by an average increment of 7–8 dB documented in the second, follow-up audiometry. These results are in line with those described by Chang et al. [2018] in their review on AAT injuries during military training.

The importance of early steroid treatment after AAT injury (within 24 h) is supported by Holy et al. [2021], showing a direct correlation between the onset of treatment and audiometric outcomes; the best outcomes were observed in patients who were treated within 24 h after AAT injury. Patients who were treated within 7 days showed better outcomes compared to those who started treatment later than 7 days after AAT injury [Holy et al., 2021]. In addition, the authors offered a “rescue” protocol including HBOT in patients who showed no audiometrical improvement after 7 days of oral steroid treatment. In these cases, HBOT demonstrated a positive effect on hearing thresholds; however, the results were inconclusive, and more research is needed on this subject. A different study that assessed the effect of early HBOT in AAT injury concluded that early initiation of HBOT (<2 days after injury) led to better outcomes, compared with late onset of treatment [Bayoumy et al., 2021].

Intense weapon noise energy is abundant at the 2- to 5-kHz region. HPDs play a major role in attenuating noise level at such frequencies, in a linear or nonlinear fashion. A nonlinear HPD attenuates peak pressures of impulse noise but allows speech and soft sound to be heard by the user. Active HPDs with embedded processors have further abilities. By constantly monitoring sound levels reaching tiny microphones embedded in the HPDs, soft sounds such as speech are enhanced while intense sound such as weapon noise is actively attenuated [Fink et al., 2019]. Improper or lack of use of HPDs in combat is dangerous and may result in temporary threshold shift or permanent threshold shift [Hecht et al., 2019].

We found that subjects with a substantial hearing loss (AC ≥35 dB) demonstrated significant improvements in BC thresholds at 3- and 4-kHz frequencies. These results are similar to those described in recent studies [Choi et al., 2019] and can be attributed to the suggested mechanism of injury to the inner ear structures. The exact pathophysiology of SNHL following AAT injury is not completely understood. Animal models demonstrated anatomic changes, such as stereocilia distortion of the inner and outer hair cells and rupture of the Reissner's membrane [Darrat et al., 2007]. At the molecular level, it was shown that excessive amounts of glutamate were released at the junction of the inner hair cells and afferent neurons in the peripheral auditory system causing ionic imbalance, leading to a massive entry of water and calcium into the afferent neuron, and eventually cell death [Duan et al., 2000; Seidman et al., 2003]. One of the theories regarding the role of steroid treatment is related to their role in decreasing intracellular edema. However, there are conflicting data in the literature regarding the efficacy of oral steroid treatment for AAT. Riemenschneider et al. [2014] investigated the otologic outcomes after the Boston Marathon bombings (2013) and found no statistically significant changes in BC thresholds between patients who received steroid treatment (a total of 8 patients) and those who were not treated with steroids, although they did notice “a clinical trend toward improvement.” Another study by Van Haesendonck et al. [2018] described the otologic outcomes after the Brussels Airport attack (2016) and showed statistically significant changes in PTA thresholds before and after oral steroid treatment. Yet, in their study, all patients received oral steroid treatment, with no comprisable control group.

The time interval from AAT injury to triage, first audiometry and referral to an otolaryngologist, is crucial to improve the prognosis of AAT injury if steroid therapy is initiated, as shown in our analysis. Yet, treatment was initiated within 24 h in only 70 of our patients, and in 200 patients, it was initiated afterward. Efforts aimed to shorten the time between the onset of AAT and the initiation of treatment to 24 h are constantly underway, raising the awareness of IDF commanders and field medical staff to identify AAT injuries and referral of any suspected AAT injuries on time to a secondary level medical facility. In a small country like Israel, we believe that it is feasible in most cases.

The advantages of our work include (1) a large cohort of previously healthy subjects after a suspected AAT injury (*n* = 263), an efficient ambulatory network that is able to provide quick audiometry and otolaryngology services for initial assessment, and flexible availability of follow-up audiometries, both conducted at the same institute. The fact that there were no definite clinical guidelines for AAT injury treatment allowed us to retrospectively examine whether there were any significant differences between the different steroid regimens, and (2) our results are generalizable also to civilian settings in our country, due to the high availability of otolaryngological and audiological services across the country.

We acknowledge some limitations: (1) our study is limited by its retrospective nature; however, randomized control trials on the subject are not ethically feasible (2) because all our study subjects were recruits of young age (18–35 years), without any substantial morbidities and contraindications to steroid therapy, and thus our findings may not be generalizable to subjects with comorbidities presenting with AAT injury; (3) HBOT, with or without concurrent steroid treatment, is increasingly being investigated as playing a major role in AAT injury treatment [Yilikoski et al., 2008; Oya et al., 2019; Holy et al., 2021]. In our study cohort, it was not part of our study protocol, and therefore we cannot comment on the efficacy of this modality; (4) we observed a tendency to prescribe oral steroid treatment to subjects presenting with more severe hearing losses, when compared to those presenting with milder losses, as documented in the first audiometry. This may lead to a selection bias. However, this disadvantage is commonly seen in other similar retrospective studies that also showed that in the absence of a consensus treatment, steroids are prescribed as the “last choice” option with no evidence-based knowledge; (5) we chose to identify the audiometric changes in the most affected ear. For example, if a subject demonstrated a mild hearing loss in his right ear but a moderate hearing loss in his left ear, we described the audiometric changes in his left ear only, for two reasons. First, we sought to identify the clinically relevant implications, and therefore we assumed that the greater improvement in the more affected ear would lead to favor a steroid treatment in the setting of an AAT injury, rather than observation only. Second, due to our large cohort, we demonstrated statistically significant results, showing improvement in the more affected ear, with no need to add the contralateral ear to the calculations. We believe that adding the contralateral ear(s) would not have contributed to the conclusions and recommendations of this work, and (6) we did not collect data regarding steroid-related side effects. Because our cohort comprised otherwise healthy subjects, with no concurrent morbidities or contraindications to steroid treatment (i.e., diabetes mellitus and hypertension), we believe that such adverse events were negligible and not clinically significant, if occurred at all.

## Conclusion

The results of this study support the accumulating evidence describing the therapeutic effect of steroid treatment in cases of AAT injury. In light of our results, we recommend a high-dose oral steroid treatment regimen in subjects after AAT injury without any known contraindications to steroid treatment. As early identification and initiation of steroid therapy is crucial in improving hearing outcomes, we recommended to conduct an audiometry within 24 h following an AAT injury, preferably. In addition, we recommend a long-term high-dose steroid regimen to be initiated at the earliest timing possible after confirmed AAT injury. Further studies should be conducted to assess the efficacy of steroid treatment in AAT injury.

## Statement of Ethics

The work has been approved by the IDF Medical Corps Institutional Review Board (Protocol No. 1929-2018) that waived the requirement for written informed consent in lieu of its retrospective study design.

## Conflict of Interest Statement

The authors have no conflicts of interest.

## Funding Sources

There was no funding for this study.

## Author Contributions

Dr. Elchanan Zloczower: author, study conception and design, and manuscript preparation. Dr. Nir Tsur: co-author, statistical analysis, and manuscript preparation. Mrs. Shir Hershkovich: data collection and interpretation. Dr. Nir Fink: data collection and interpretation. Dr. Tal Marom: senior author, study conception and design, and manuscript preparation. All authors have read and approved the final version of the manuscript.

## Data Availability Statement

All data generated or analyzed during this study are included in this article. Further inquiries can be forwarded to the corresponding author.

## Figures and Tables

**Table 1:** Demographic and clinical data by treatment group

	Nonsteroid treatment	Steroid treatment	*p* value (<0.05)
Subjects, *n* (%)	126 (48)	137 (52)	
Males, *n* (%)	106 (84.1)	126 (92)	0.057
Mean age ± SD, years	19.99±2.40	20.30±2.38	0.314
Time schedule, days			
Time from injury to first audiometry	5.4±5.25	4.79±7.86	0.467
Time from first audiometry to physician	7.75±7.4	3.86±3.75	**<0.0001**
Presenting symptoms, *n* (%)			
Hearing loss	75 (59.5)	99 (72.3)	**0.037**
Tinnitus	96 (76.2)	101 (73.7)	0.671
Hyperacusis	35 (27.8)	53 (38.7)	0.068
Aural fullness	35 (27.8)	46 (33.6)	0.35
Otalgia	49 (38.9)	56 (40.9)	0.801
Dizziness	15 (11.9)	19 (13.9)	0.714
Others	3 (2.3)	11 (8.0)	**0.041**
Noise exposure setting, *n* (%)			
Open	90 (71.4)	100 (73)	0.465
Closed	32 (25.4)	29 (21.2)	−
Mixed	1 (0.8)	0 (0)	−

**Table 2:** Distribution of hearing impairment

	Nonsteroid treatment (*N* = 252), *n* (%)	Steroid treatment (*N* = 274), *n* (%)	*p* value (<0.05)
Hearing impairment during initial evaluation			
Normal hearing*	225 (89.3)	228 (83.3)	
Mild hearing loss**	22 (8.7)	38 (13.9)	0.194
Moderate hearing loss***	5 (2.0)	7 (2.6)	
Moderate to severe hearing loss****	0 (0.0)	1 (0.4)	
Overall improvement^a^	54 (21.4)	83 (30.3)	**0.02**
A hearing improvement ≥20 dB	2 (0.8)	7 (2.6)	0.11
Normal hearing after treatment	242 (96.0)	262 (95.6)	0.81
Improved from mild hearing loss to normal	19 (7.5)	32 (11.7)	0.1

* Defined as pure tone average for air conduction <25 dB.

** Defined as pure tone average for air conduction >25 dB and <40 dB.

*** Defined as pure tone average for air conduction >40 dB and <55 dB.

**** Defined as pure tone average for air conduction >55 dB and <70 dB.

a Defined as second air conduction pure tone average < first air conduction pure tone average.

**Table 3:** Audiometry results by treatment group

	Nonsteroid treatment (*N* = 252)	Steroid treatment (*N* = 274)	*p* value (<0.05)
First PTA BC average ± SD, dB	12.98±6.53	16.7±9.38	**<0.0001**
Second PTA BC average ± SD, dB	10.98±5.97	12.5±6.72	0.102
First air conduction, dB			
6,000 Hz, average ± SD	25.41±16.54	33.91±21.37	**<0.0001**
8,000 Hz, average ± SD	24.9±18	31.37±22.47	**<0.0001**
First bone conduction, dB			
3,000 Hz, average ± SD	18.49±11.17	27.57±17.4	**<0.0001**
4,000 Hz, average ± SD	21.06±13.11	32.1±17.74	**<0.0001**
Second air conduction, dB			
6,000 Hz, average ± SD	18.17±11.91	21.01±15.61	**0.019**
8,000 Hz, average ± SD	17.72±14.60	19.39±16.82	0.226
Second bone conduction, dB			
3,000 Hz, average ± SD	14.52±10.11	20.21±13.55	**0.001**
4,000 Hz, average ± SD	17.65±11.14	23.88±14.62	**0.01**
Air conduction difference, dB			
6,000 Hz, average ± SD	7.24±13.35	13.05±16.26	**<0.0001**
8,000 Hz, average ± SD	7.18±14.07	12.1±16.84	**<0.0001**
Bone conduction difference, dB			
3,000 Hz, average ± SD	4.73±9.75	10.18±13.93	**0.002**
4,000 Hz, average ± SD	3.9±10.48	11.37±14.5	**<0.0001**

BC, bone conduction.

**Table 4:** Audiometry results by onset of treatment

	<24-h treatment initiation (*N* = 70)	>24-h treatment initiation (*N* = 200)	*p* value (<0.05)
First audiometry AC, dB			
6,000 Hz, average ± SD	31.57±21.78	34.7±21.38	0.295
8,000 Hz, average ± SD	30.57±22.5	31.5±22.65	0.768
First audiometry BC, dB			
3,000 Hz, average ± SD	30.88±20.75	26.85±16.44	0.265
4,000 Hz, average ± SD	31.75±18.45	32.3±17.76	0.866
Second audiometry AC, dB			
6,000 Hz, average ± SD	17.17±13.9	22.53±16.05	**0.009**
8,000 Hz, average ± SD	16.3±15.83	20.68±17.15	0.064
Second audiometry BC, dB			
3,000 Hz, average ± SD	18.13±10.82	20.98±14.15	0.064
4,000 Hz, average ± SD	16.04±9.32	26.15±15.02	**<0.001**
AC difference, dB			
6,000 Hz, average ± SD	14.711±17.57	12.26±15.82	**0.283**
8,000 Hz, average ± SD	14.57±17.38	10.88±16.46	**0.115**
BC difference, dB			
3,000 Hz, average ± SD	12.82±17.11	9.42±13.05	0.382
4,000 Hz, average ± SD	13.48±15.18	10.76±14.42	0.43

AC, air conduction; BC, bone conduction.

**Table 5:** Audiometry results by duration of treatment

	Treatment duration <7 days (*N* = 192)	Treatment duration ≥7 days (*N* = 56)	*p* value (<0.05)
First audiometry AC, dB			
6,000 Hz, average ± SD	33.51±21.3	36.18±22.79	0.268
8,000 Hz, average ± SD	31.21±22.53	31.58±23.16	0.925
First audiometry BC, dB			
3,000 Hz, average ± SD	27.16±17.38	30.86±18.32	0.3
4,000 Hz, average ± SD	31.47±17.44	35.69±19.85	0.247
Second audiometry AC, dB			
6,000 Hz, average ± SD	20.09±14.57	27.63±20.19	**0.032**
8,000 Hz, average ± SD	18.78±16.71	24.21±17.45	**0.067**
Second audiometry BC, dB			
3,000 Hz, average ± SD	18.51±12	28.41±16.78	0.015
4,000 Hz, average ± SD	21.82±12.38	33.41±19.23	0.012
AC difference, dB			
6,000 Hz, average ± SD	13.61±16.88	8.55±11.26	**0.076**
8,000 Hz, average ± SD	12.57±17.06	7.37±14.18	0.076
BC difference, dB			
3,000 Hz, average ± SD	10.92±14.77	7.05±9.96	**0.15**
4,000 Hz, average ± SD	12.1±14.7	8.41±13.92	0.292

AC, air conduction; BC, bone conduction.

## Notes

Elchanan Zloczower and Nir Tsur equally contributed to the study.
